# In Vitro Studies on the Trafficking of Dendritic Cells Through Endothelial Cells and Extra-Cellular Matrix

**DOI:** 10.1155/2000/39893

**Published:** 2000

**Authors:** Giancarlo Bianchi, Giovanna D'amico, Luisella Varone, Silvano Sozzani, Alberto Mantovania, Paola Allavena

**Affiliations:** 1 lstituto di Ricerche Farmacologiche Mario Negri Via Eritrea 62 Milan 20157 Italy; 2 Dept. Biotechnology Section of General Pathology University of Brescia Italy

**Keywords:** dendritic cells, chemokines, chemotaxis, receptors, transendothelial migration, endothelial cells

## Abstract

Dendritic cells (DC) are antigen presenting cells (APC) with the unique ability to initiate an immune response. Immature DC are localized in peripheral tissues where they exert a sentinel function for incoming antigens (Ag). After Ag capture and exposure to inflammatory stimuli DC undergo maturation and migrate to regional lymph nodes where the presentation of antigenic peptides to T lymphocytes takes place. Thus their correct functioning as APC involves localization in tissues and trafficking via the lymph or blood to lymphoid organs. In the present study we have investigated the ability of DC to interact in vitro with human vascular endothelial cells (EC) and extracellular matrix (ECM). DC are differentiated from monocytes by in vitro exposure to GM-CSF and IL-13 for 7 days. In adhesion assays a considerable proportion of DC binds to resting EC monolayers and this adhesion is inhibited by anti-CD11a and CD11b, but not anti-CD11c mAbs. Binding to a natural ECM, derived from cultured EC involves VLA-4 and VLA-5 integrins. In a transmigration assay, 10 % of input cells are able to cross the EC monolayer in the absence of exogenous stimuli. The amount of DC transmigrated through a monolayer of EC was increased of 2-3 fold by C-C chemokines RANTES, MIP1α, and MIP-1β. Most importantly, in view of the trafficking pattern of these cells, a significant proportion of DC can migrate in a reverse transmigration assay, i.e. across the endothelial basement membrane and subsequently, across endothelial cells. Upon exposure to immune or inflammatory signals peripheral DC undergo maturation and migration to lymphoid organs. Functional maturation is associated with loss of responsiveness to chemokines present at sites of inflammation (e.g. MIP1α, MIP1β and RANTES) and acquisition of a receptor repertoire which renders these cells responsive to signals which guide their localization in lymphoid organs (e.g. MIP3β). A better understanding of the molecular basis of DC trafficking may provide molecular and conceptual tools to direct and modulate DC localization as a strategy to upregulate and orient specific immunity.


*Developmental Immunology*, 2000, Vol. 7(2-4), pp. 143-153
Reprints available directly from the publisher
Photocopying permitted by license only

© 2000 OPA (Overseas Publishers Association)
N.V. Published by license under the
Harwood Academic Publishers imprint,
part of the Gordon and Breach Publishing Group.
Printed in Malaysia

### In Vitro Studies on the Trafficking of Dendritic Cells Through Endothelial Cells and Extra-Cellular Matrix

GIANCARLO BIANCHI[a], GIOVANNA D'AMICO[a], LUISELLA VARONE[a], SILVANO SOZZANI[a], ALBERTO MANTOVANI[ab] and PAOLA ALLAVENA[a*]

[a]*Istituto di Ricerche Farmacologiche Mario Negri, Via Eritrea 62, 20157 Milan and* [b]*Dept. Biotechnology, Section of General Pathology, University of Brescia, Italy*

Dendritic cells (DC) are antigen presenting cells (APC) with the unique ability to initiate an immune response. Immature DC are localized in peripheral tissues where they exert a sentinel function for incoming antigens (Ag). After Ag capture and exposure to inflammatory stimuli DC undergo maturation and migrate to regional lymph nodes where the presentation of antigenic peptides to T lymphocytes takes place. Thus their correct functioning as APC involves localization in tissues and trafficking via the lymph or blood to lymphoid organs. In the present study we have investigated the ability of DC to interact in vitro with human vascular endothelial cells (EC) and extracellular matrix (ECM). DC are differentiated from monocytes by in vitro exposure to GM-CSF and IL-13 for 7 days. In adhesion assays a considerable proportion of DC binds to resting EC monolayers and this adhesion is inhibited by anti-CD11a and CD11b, but not anti-CD11c mAbs. Binding to a natural ECM, derived from cultured EC involves VLA-4 and VLA-5 integrins. In a transmigration assay, 10 % of input cells are able to cross the EC monolayer in the absence of exogenous stimuli. The amount of DC transmigrated through a monolayer of EC was increased of 2–3 fold by C-C chemokines RANTES, MIP1α, and MIP-1β. Most importantly, in view of the trafficking pattern of these cells, a significant proportion of DC can migrate in a reverse transmigration assay, i.e. across the endothelial basement membrane and subsequently, across endothelial cells. Upon exposure to immune or inflammatory signals peripheral DC undergo maturation and migration to lymphoid organs. Functional maturation is associated with loss of responsiveness to chemokines present at sites of inflammation (e.g. MIP1α, MIP1β and RANTES) and acquisition of a receptor repertoire which renders these cells responsive to signals which guide their localization in lymphoid organs (e.g. MIP3β). A better understanding of the molecular basis of DC trafficking may provide molecular and conceptual tools to direct and modulate DC localization as a strategy to upregulate and orient specific immunity.

*Keywords:* dendritic cells, chemokines, chemotaxis, receptors, transendothelial migration, endothelial cells

*Abbreviations:* DC, dendritic cells, mono-DC, DC derived from monocytes, CD34-DC, DC derived from CD34+ stem cells, APC, antigen presenting cell, LC, Langerhans cells, MLR, Mixed Leukocyte Reaction, EC, endothelial cells, ECM, extracellular matrix, MIP, macrophage inflammatory protein, RANTES, regulated on activation normal T cell expressed and secreted, SLC, secondary lymphoid tissue chemokine

* Corresponding Author.

### INTRODUCTION

Dendritic cells (DC) are professional antigen presenting cells (APC) which play a key role in the initiation of an immune response. DC precursors migrate from the bone marrow via the blood stream to non lymphoid tissues to become resident immature DC (Steinman, 1991; Hart, 1997; Banchereau et al. 1998; Sallusto et al. 1999). DC at the immature stage of differentiation efficiently take up and process incoming antigens (Ag). Upon exposure to immune or inflammatory signals peripheral DC undergo maturation and migrate as veiled cells to the T cell areas of lymph nodes (Steinman, 1991; Austyn, 1996; Hart, 1997; Banchereau et al. 1998; Sallusto et al. 1999).

This trafficking of DC to lymph nodes is critical to their overall function as APC, in fact it is established that surgical removal of afferent lymphatic vessels prevents sensitization to skin transplants or to contact allergens (Frey et al. 1957; Barker et al. 1968). During their migration DC undergo phenotypical and functional maturation. They stop capturing antigens and upregulate the expression of co-stimulatory molecules (CD54, CD58, CD80, and CD86). After reaching the lymph node subcapsular sinus, DC migrate to the T cell areas. These DC, identified as interdigitating DC, have a strong ability to present the antigen to naive T lymphocytes. The homing to lymphoid organs and the activation of T cells is apparently the final goal of DC biological program since, very likely, they undergo apoptosis within the T cell areas.

DC have long been recognized as highly motile cells. In vivo studies have demonstrated that DC use specific pathways of tissue trafficking. Application of a fluorescent marker to skin induce migration of Langerhans cells (LC) and a day later fluorescent LC are found in the draining lymph nodes (Macatonia et al. 1987). It has also been described that inhaled pathogens induce a very rapid mobilization of DC in the airway epithelium (McWilliam et al. 1994; McWilliam et al. 1996). After i.v. injection of inert particles, cells with DC characteristic are recruited to the hepatic sinusoid and then translocate to the hepatic lymph (Matsuno et al. 1996). Inflammatory signals exert a dual influence on the migration of DC. On the one hand increased recruitment of blood precursors is observed at sites of infection or inflammation. On the other, systemic treatment with LPS provokes a massive egress of DC from several organs, such as skin, heart, kidney, intestine and cause DC migration from marginal zone of the spleen to T cell areas (Macpherson et al. 1995; Roake et al. 1995; De Smedt et al. 1996).

The complex trafficking of DC involves their interaction with the endothelial linings and with extracellular matrix (ECM). In this study we will discuss how adhesion molecules and chemokines regulate the interaction of DC with endothelial cells (EC) and with ECM, and will analyze which chemotactic signals regulate the migratory behaviour of DC at different stages of maturation.

### RESULTS

### In vitro adhesion of DC to EC monolayers and ECM

DC were differentiated in vitro from highly purified human monocytes in the presence of GM-CSF+ IL-13 for 7 days. As previously reported these cultured DC expressed high levels of CD1a, MHC Class II and CD86 and were strong stimulators of allogeneic T lymphocyte proliferation in Mixed Leukocyte Reaction (MLR) (Piemonti et al. 1995; Sozzani et al. 1997). In previous experiments we showed that DC expressed high levels of β2 integrins and, among β1 integrins, CD49d and CD49e (VLA-4 and VLA-5). All the cells stained positive for CD61 (β3), for CD31 (PECAM-1) and were negative for CD62L (L-selectin, data not shown) (D'Amico et al. 1998).

The identification of adhesion molecules involved in the binding to EC and to ECM was performed by using functional blocking mAb. A representative experiment is shown in Fig. 1: anti-CD11a, anti-CD11b and anti-CD18 mAbs partially inhibited adhesion (52%, 64%, and 68% respectively, while anti-CD11c, although expressed to a very high extent on DC, was not involved in binding to endothelium

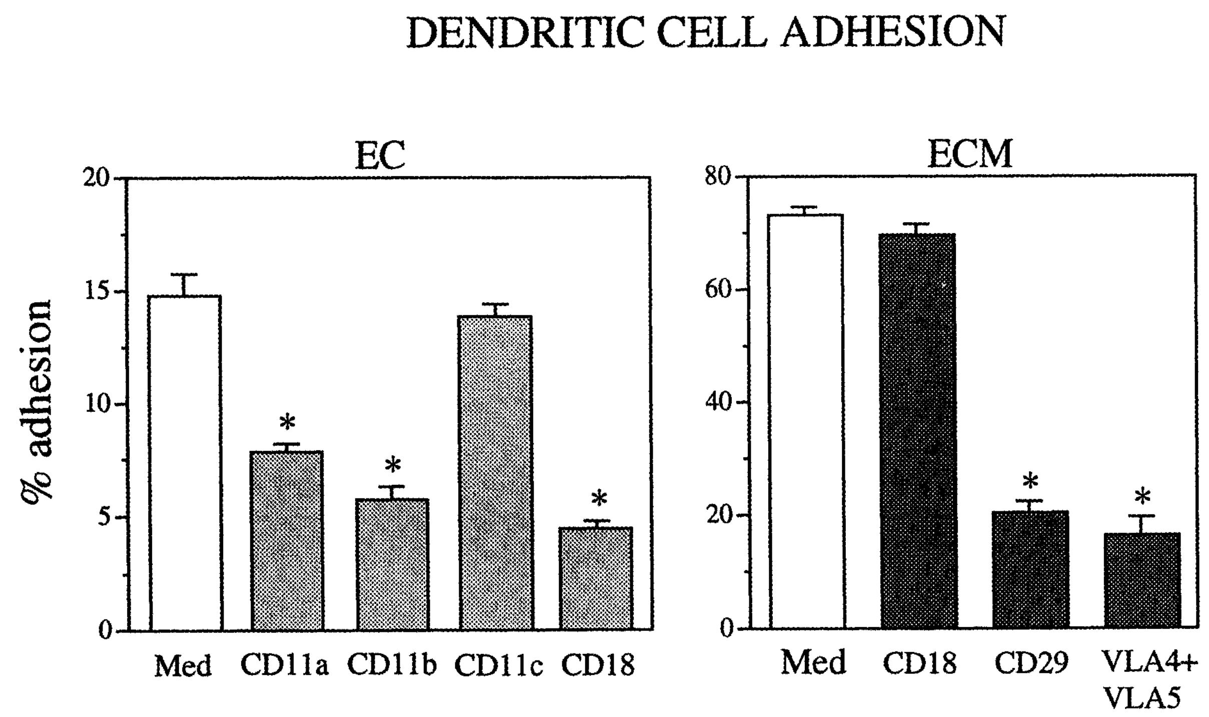

FIGURE 1 Molecules involved in the adhesion of DC to resting EC (A) and to EC-derived matrix (ECM) (B). DC were differentiated from blood monocytes by 7-day culture with 50 ng/ml GM-CSF and 10 ng/ml IL-13. $^{51}$Cr-labeled DC were preincubated for 20 min at room temperature with the indicated mAb, thereafter DC were co-incubated for 1h at 37°C. Results are expressed as % of adherent cells, mean±SE of three replicates. ECM was prepared by culturing a monolayer of EC on policarbonate filters for at least 5 days; EC were then stripped by a NH4OH + Triton x-100 solution for 30 sec. (*) Statistically significant at $p<0.01$ vs medium control

(Fig. 1A). These results were confirmed in a larger series of experiments (mean inhibition with anti-CD18, 61±4% mean±SE of 7 experiments). Binding of DC to a natural ECM, derived from cultured EC, was usually higher than to EC monolayers: 52±6% of input cells compared to 17±4 % (mean±SE of 6–8 experiments). Anti-VLA-4+VLA-5 mAbs or anti-CD29 inhibited most of the binding (>75% inhibition) while – as expected – antiCD18 had no effect, Fig. 1B).

### Direct and reverse transendothelial migration

As mentioned above, blood DC precursors, like other leukocytes, exit from the blood stream by interacting with the endothelial cell barrier from the apical side. However, when peripheral DC become activated, and migrate from the tissue of residence to afferent lymphatics, they first interact with the basement membrane of lymphatic or blood vessels and then with the apical side. We therefore established an assay to mimic both the apical/basal transmigration (direct) as well as the basal/apical transmigration (reverse). These methods were previously described in details (Bianchi et al. 1993; D'Amico et al. 1998). As shown in Fig. 2 DC effectively migrated through resting EC monolayers in a direct transmigration assay (left panel). The proportion of transmigrated cells corresponds to 10.6±1.6% of input cells and to 33% of adherent cells (mean±SE; n=6).

In the reverse transmigration assay, an upper filter is coated with ECM and a lower filter is coated with a monolayer of EC, placed upside down. The mean amount of transmigrated cells relative to input was

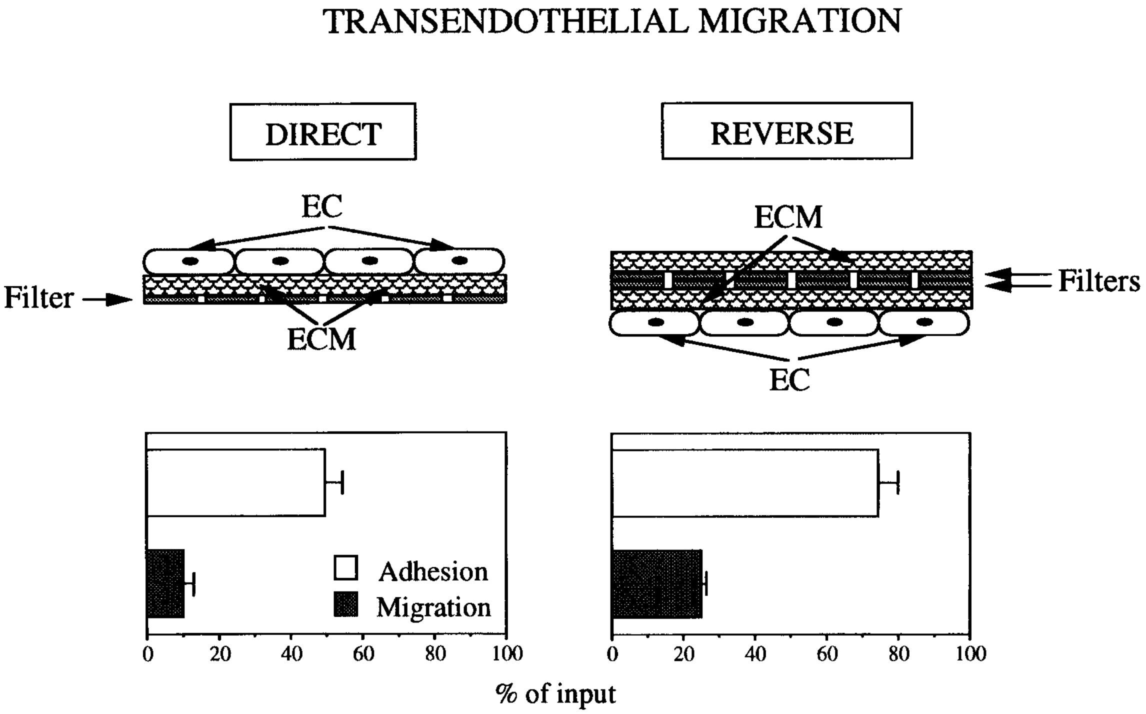

FIGURE 2 Trans-endothelial migration of DC. In the direct transmigration (A), a porous policarbonate filter was coated with a monolayer of EC and placed in modified Boyden chamber. In the reverse transmigration (B) a double filter system was used. The upper filter was coated with an ECM and the lower filter was coated with a monolayer of EC placed upside down. $^{51}$-Cr-labeled DC were seeded in the upper compartment of the chamber and co-incubated with EC monolayer for 1h at 37°C. Results are expressed as % of adherent and transmigrated cells mean±SEM of three replicates

13±4% (n = 4) which corresponds to 20.8% of adherent cells. The reason for a lower proportion of transmigrated-over-adherent cells in the reverse assay resides probably in the fact that the double filter is a thicker barrier to cross.

### Adhesion and transmigration of immature and mature DC

Upon exposure to microbial products (e.g. LPS), inflammatory cytokines (e.g. IL-1 and TNF) or immune stimuli (e.g. CD40 ligation), peripheral DC undergo maturation and migrate as veiled cells to the T cell areas of lymph nodes (Steinman, 1991; Austyn, 1996; Hart, 1997; Banchereau et al. 1998; Sallusto et al. 1999). DC at the two stages of differentiation display different functional activities: mature DC have low Ag uptake but are potent APC, while immature DC are poor APC but have a strong activity to uptake and process Ags. As it is not clear the precise stage of differentiation/maturation of the migrating cell, it was of interest to compare the migratory ability of mature and immature DC. Fig. 3 shows that mature DC migrate with less efficiency through an endothelial monolayer, compared to immature DC, while the adhesive ability was similar. Overall the spontaneous ability of mature DC to cross the endothelial barrier was half that of immature cells.

### Trans-endothelial migration in response to chemokines

The above described lower migratory activity of mature DC could be due to an unresponsiveness to

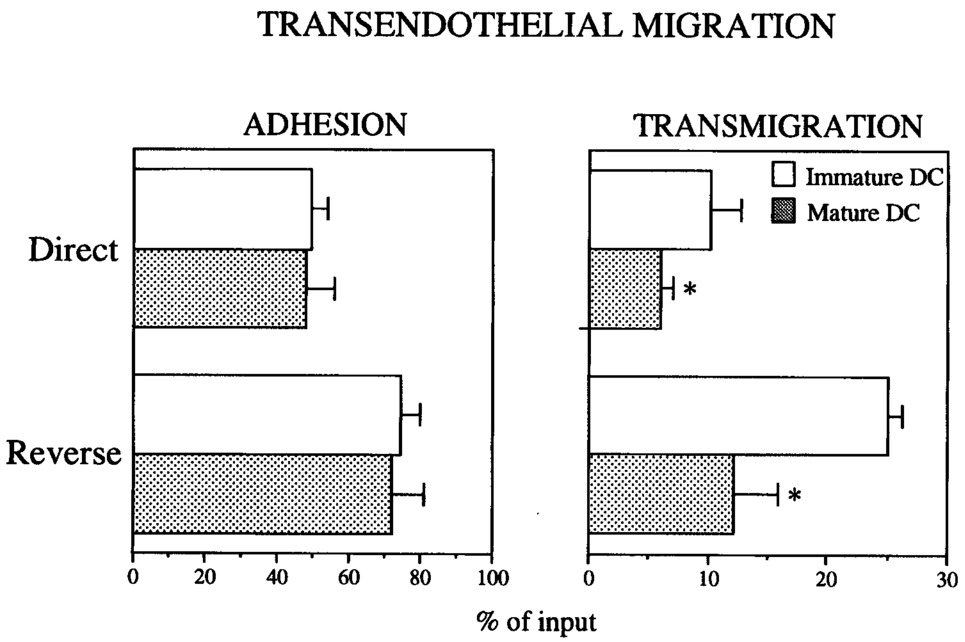

FIGURE 3 Effect of maturation on DC trans-endothelial migration. To induce maturation, immature DC were incubated with CD40L-transfected cells (CD40L) (5:1 ratio), for 48 h before being tested. $^{51}$Cr-labeled DC were seeded in the upper compartment. Radioactivity present in the lower compartment was evaluated after incubation at 37°C for 1 h chemotactic factors which are known to regulate the directional migration of these cells and are produced by EC (Sozzani et al. 1999; Sallusto et al. 1999). We therefore tested mature and immature DC in a transmigration assay in the presence of chemokines. Fig. 4 shows that MIP1α, MIP3β and RANTES placed in the lower compartment of the chemotactic chamber, significantly attracted immature DC more than medium control. Surprisingly, mature DC do not respond any more to MIP-1α, RANTES and MIP-1β. In marked contrast, the response to MIP3β was much higher than that of immature DC. These findings are in line with the expression of chemokine receptors in immature and mature DC, as recently reported (Dieu et al. 1998; Sozzani et al. 1998; Sallusto et al. 1998; Yoshida et al. 1997; Lin et al. 1998). In fact, as summarized in Table I, maturation of DC is associated with switch of chemokine receptor expression. Specifically, mature DC downregulate CCR1 and CCR5 (receptors for MIP1α, MIP1β and RANTES) and upregulate CCR7 (MIP3β and SLC).

### Differentiation of DC on ECM-conditioned tissue culture plastic

As the stage of differentiation and maturation of DC play such an important role in the functional activity of DC, it was of interest to assess environmental factors which may modulate the in vitro differentiation of monocytes into DC. We therefore asked the question whether culture of monocytes on a substrate other than plastic could favour, or inhibit, the process of DC differentiation. To verify this, monocytes were incubated on ECM-coated tissue culture plates or standard plates. ECM was prepared as described above, by stripping a monolayer of EC. Fig. 5 shows that the phenotype of DC differentiated on

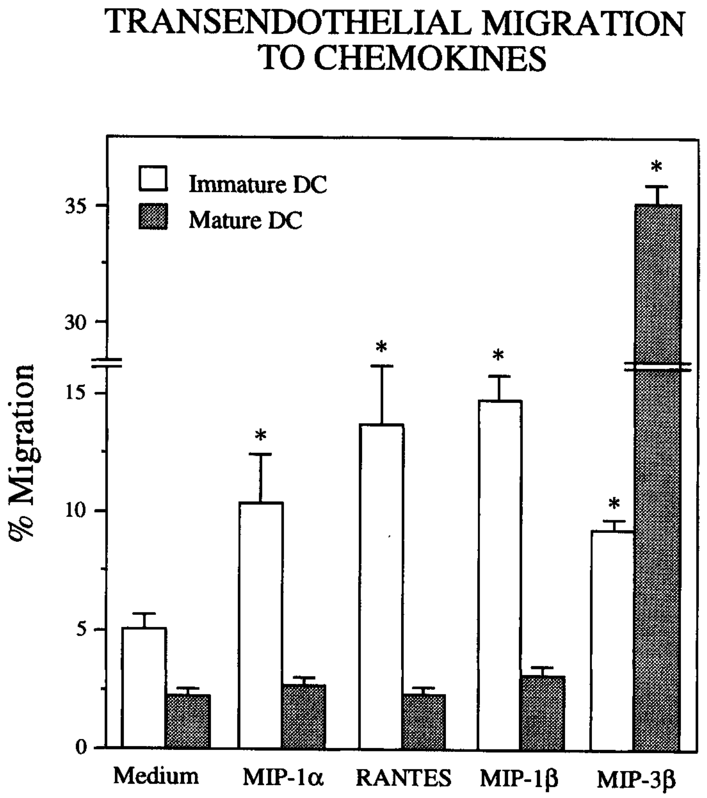

FIGURE 4 Effect of chemokines on transendothelial migration of immature and CD40L-matured DC. Policarbonate transwell inserts were coated with a monolayer of EC and chemoattractants (100ng/ml) were seeded in the lower compartment. 51-Cr labeled DC were placed in the upper compartment and incubated for 1h at 37°C. * Statistically significant at p<0.01

ECM-coated plastic did not differ from that of standard DC cultures. Both cell types had high levels of CD1a. The expression of the co-stimulatory molecule CD86, which is low in immature DC, was also similar, indicating that the stage of differentiation/maturation in the two culture systems was not significantly different. Finally, also CD68, a marker highly expressed in mature macrophages was similarly low, suggesting that incubation on ECM does not favour the differentiation of monocytes into macrophages. DC cultured on ECM were also tested for their accessory function in a MLR assay. These DC were high APC for allogenic T cells, with no significant difference compared to standard DC (data not shown).

TABLE I Switch of chemokine receptor expression during DC maturation

| *Receptors* | *Immature D C* | *Mature DC* | *Ligands* |
|---|---|---|---|
| **CCR1** | + | - | MIP-1α,[a] RANTES MCP 2/3 |
| **CCR5** | + | - | MIP-1α, MIP-1β, RANLES, MCP-2 |
| **CCR7** | +/- | ++ | MIP-3β, SLC |

a. MIP: macrophage inflammatory protein; RANTES: regulated on activation normal T cell expressed and secreted;MCP: monocyte chemotactic protein; SLC: secondary lymphoid tissue chemokine.

### DISCUSSION

Trafficking is a crucial component of the immunobiology of DC. Technical improvements, including appropriate culture techniques and the setting up of convenient assays representative of different steps of trafficking, have provided the tools to begin the dissection of the molecular basis of this complex series of events.

We have shown that even in the absence of exogenously applied stimuli, DC bind to EC and more consistently to ECM and efficiently cross these barriers (D'Amico et al. 1998). Similar results on transendothelial migration of human monocyte-derived DC were reported (Jancic et al. 1998; Lin et al. 1998). Interestingly, we also showed that DC perform a reverse transmigration, i.e. from the basal to the apical direction, a situation that is most likely to occur in vivo when maturing DC migrate fromperipheral tissues to regional lymph nodes (D'Amico et al. 1998).

Another model of reverse transmigration was described by Muller et al with blood monocytes, which are DC precursors. In this assay EC were grown on collagen type I. Blood monocytes were allowed to penetrate into the collagen gel beneath the EC monolayer and, subsequently, most of them, cross again the overlying endothelium in 24–48 h. A major issue raised by these Authors is that reverse transmigration combined with phagocytosis of particles in the sub-endothelial collagen induced maturation of DC (Randolph et al. 1998). Binding to collagen typeI – but not fibronectin – stimulated maturation of DC, most likely by increasing the TNF production by DC themselves (Brand et al. 1998). In contrast, we did not find any significant positive or negative effect of ECM on the differentiatation of monocytes into DC.

Chemokines have emerged as major regulators of DC migration in vitro and in vivo (Baggiolini et al. 1997; Rollins, 1997; Mantovani et al. 1998). We and others have previously reported that DC respond to a defined set of inflammatory chemokines, including MIP1α and MIP1β, MCP3, RANTES, MDC and MIP3α (Sozzani et al. 1995; Sozzani et al. 1997; Godiska et al. 1997; Power et al. 1997; Greaves et al. 1997). More recently, it has become clear that only immature cells are responsive to the above panel of chemokines. In fact, maturation of DC is associated with a switch of chemokine receptor expression on the cell membrane. Downregulation of CCR1 and CCR5, and of CCR6 on CD34-derived DC, explains this unresponsiveness. Moreover, upregulation of CCR7 explains the increased response to its ligands MIP3b and SLC (Dieu et al. 1998; Sozzani et al. 1998; Sallusto et al. 1998; Yoshida et al. 1997; Lin et al. 1998). These results have been obtained both in classical chemotaxis assays as well as in transendothelial migration assays (Sozzani et al. 1998; Lin et al. 1998). Overall these results suggest that the unresponsiveness to inducible chemokines (MIP1α. MIP1β, RANTES etc) usually present at the inflammatory site, may favour the release of DC from peripheral tissues and their floating towards lymph nodes. Concomitantly, the increased response to MIP3β and SLC, two chemokines constitutively expressed in secondary lymphoid organs (Willimann et al. 1998; Ngo et al. 1998; Dieu et al. 1998), will guide DC directly at the right place (Sozzani et al. 1999; Cyster, 1999; Sallusto et al. 1999).

The understanding of the role of chemokines in DC trafficking in vivo has received great advantage from the use of gene modified animals. Mice homozygous for an autosomal recessive mutation, paucity of lymph node T cells (plt), fail to express SLC and have reduced MIP-3β. DC from these mice fail to accumulate in the spleen and T cell areas of lymph nodes (Gunn et al. 1999). Expression of SLC and MIP3β is also reduced in mice deficient of TNF or lymphotoxin α/β. These mice show alteration in the architecture of lymphoid organs and lymphocytes fail to adequately compartimentalize in B and T cell areas. Lymph nodes from lymphotoxin α/β-deficient mice also have reduced numbers of DC (Ngo et al. 1999), and TNF receptor 1-deficient mice have depressed migration of LC to lymph nodes (Wang et al. 1997). A role for CCR7 and its ligands in DC migration from TNF-treated skin explants was also recently indicated by the use of blocking antibodies (Saeki et al. 1999). These findings indicate that a "tonic" production of lymphotoxin α/β and TNF very likely regulate the production of chemokines by stromal cells. These

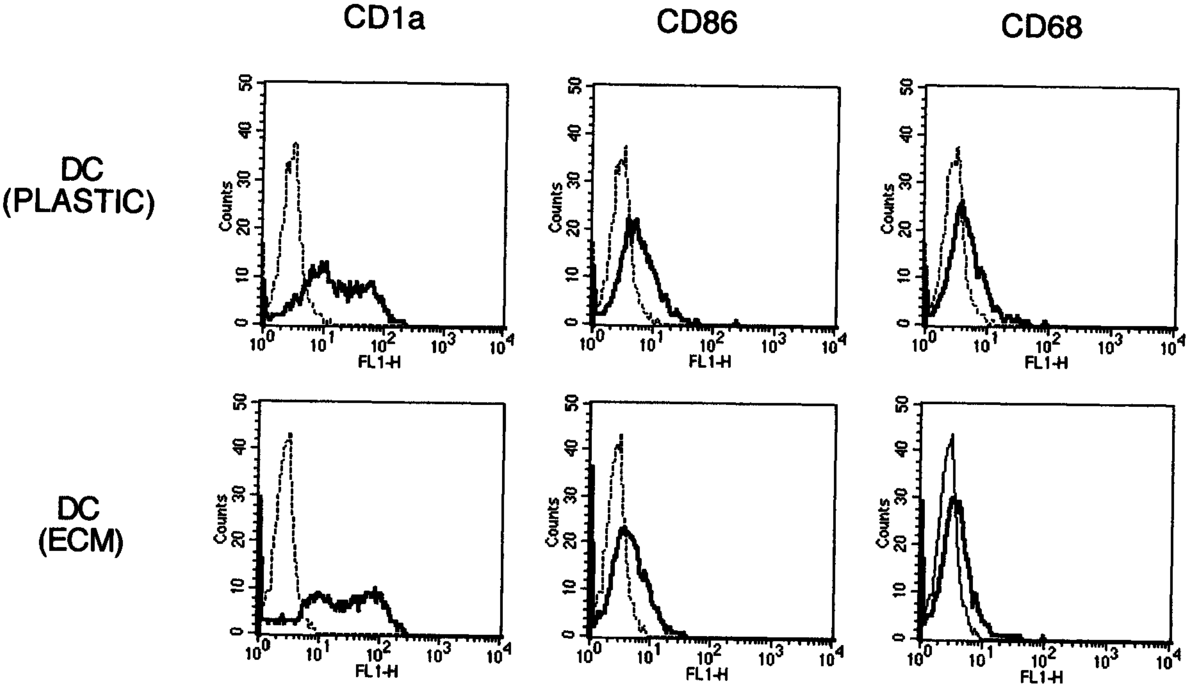

FIGURE 5 Flow cytometric analysis of membrane molecules expressed by DC differentiated on ECM-coated tissue culture plates. Monocytes were cultured with 50 ng/ml GM-CSF and 20 ng/ml IL-13 for 7 days on standard tissue culture plates or on ECM-coated plates prepared by stripping a monolayer of EC (see M&M). Cells were labeled with the designed mAb and then with FITC-labelled goat anti mouse-lg chemokines, only apparently produced in a constitutive way, are crucial for the recruitment of lymphoid cells and APC from the periphery to the lymph node.

DC are considered promising tools and targets of immunotherapeutic strategies; a better understanding of the molecular basis of DC trafficking may provide molecular and conceptual tools to direct and modulate DC traffic as a strategy to upregulate and orient specific immunity.

### MATERIALS AND METHODS

### Culture media and reagents

The following reagents were used for separation of effector cells, cell culture and experimental assays: pyrogen-free saline for clinical use and distilled water (Bieffe, Sondrio, Italy); medium RPMI 1640 (10X concentrated, Biochrom KG, Berlin, Germany); medium 199 (Gibco, Paisley, UK); glutamine (Gibco); penicillin and streptomicin (Gibco); aseptically collected FCS (Hyclone, Logan, UT). The routinely employed tissue culture medium was RPMI 1640 with 2 mM glutamine, 50 mg/ml of gentamicin, 10% FCS.

### Cytokines

Human recombinant GM-CSF, was a generous gift from Novartis (Basel, Switzerland). Human recombinant IL-13 was a kind gift from Dr. A. Minty (Sanofi Elf Bio Recherches, Labège, France). Human MIP-1α, MIP-3β and RANTES, were from PeproTech Inc. (Rocky Hill, NJ, USA). Cytokines were endotoxin free as assessed by Limulus Amebocyte assay. Lipopolysaccharide (LPS; E. coli 055:B5) was from Sigma (St. Louis, MO).

### Preparation of DC cultures

DC were differentiated in vitro as previously described (Sozzani et al. 1995; Piemonti et al. 1995). Highly enriched blood monocytes (> 95% CD14+) were obtained from buffy coats by Ficoll and Percoll gradients and purified by panning on CD6-coated plastic dishes. Monocytes were cultured for 7 days at $1 \times 10^6$/ml in 6-well multiwell tissue culture plates (Falcon, Becton Dickinson, New Jersey) in RPMI (Biochrom, Berlin, FRG) 10% FCS (Hyclone, Logan, UT) supplemented with 50 ng/ml GM-CSF and 20 ng/ml IL-13. We previously described that monocyte-derived DC generated in the presence of GM-CSF+IL-13 are morphologically and functionally identical to DC cultured with GM-CSF+IL-4 (Sozzani et al. 1995; Piemonti et al. 1995). Where specified, DC were further cultured in the presence of 10 ng/ml LPS for 48 hours or co-cultured with J558L cells transfected with the gene encoding CD40L (Cella et al. 1996) (kindly provided by Dr. Peter Lane, Basel Institute for Immunology, Switzerland). DC cultured with LPS or activated by CD40 ligation for 48 hours expressed typical maturation markers and high APC activity. For instance, after CD40L activation, DC were >70% CD83+: > 70% CD80+; MHC class II bright (mean channel fluorescence > 900); and highly effective in MLR (e.g. at 3% APC T cell ratio, $^3$HTdr uptake was > 70,000 cpm; S.I. >35). Preliminary experiments confirmed that incubation of DC with the J558 mock cell line did not induce cell maturation.

### Monoclonal antibodies

The following mAbs were used: L243 (IgG2a, anti MHC class II) purchased from ATCC (Rockville, MD, USA); NA 1/34 (IgG2a, anti CD1a) Dako Denmark); TS1/22 (IgG1, anti CD11a) and TS1/18 (IgG1, anti CD18) from ATCC (Rockville, MD, USA);; 44a (IgG2a, anti CD11b) was a kind gift of Dr. R. Todd (Ann Arbor, MI,USA); L29 (IgG1 anti CD11c) was a kind gift of Dr. L. Lanier (DNAX Palo Alto, CA, USA); HP2/1 (IgG1 anti-VLA4 was a kind gift of Dr. F. Sanchez-Madrid (University of Madrid, Spain); M-A251 (IgG1 anti-CD25), IT2.2 (IgG2b anti-CD86) and IIA1(IgG1 anti VLA5) were from Pharmingen – Becton Dickinson, (NJ,USA). For FACS Analysis, cell staining with primary mAb was followed by FITC-conjugated affinity purified, isotype-specific goat anti-mouse antibodies (Techno-Genetics). Results are expressed as % of positive cells and as relative fluorescence intensity (RFI), calculated according to the formula: RFI=mean fluorescence (sample)-mean fluorescence (control) / mean fluorescence (control).

In adhesion or transmigration assays, optimally diluted mAb were preincubated with the cells for 20 min before plating; medium control contained an isotype-matched irrelevant mAb.

### Preparation of endothelial cells (EC)

Human EC were obtained from umbilical vein and cultured as previously described (Sironi et al. 1989). Routinely, we used confluent cells ($10^5$/cm$^2$ culture well) between the first and fourth passage maintained in 199 medium with 20% bovine serum (Hyclone Lab.) supplemented with endothelial cell growth supplement (50 μg/ml, Collaborative Research Inc., Lexington, MA, USA) and heparin (100 μg/ml, Sigma Chemical Co., St. Louis, MO, USA). The purity of EC cultures was checked by expression of von Willebrand factor and found to be >99% positive.

### Preparation of extra-cellular matrix (ECM)

The ECM was prepared by growing a monolayer of EC on filters for at least 5 days; EC were then stripped away by a short treatment with a 20 mM NH4OH solution + 0.5% Triton X-100 for 30 sec.

### Adhesion assay

Adhesion of DC to endothelial cells (EC) was studied as described previously (Allavena et al. 1991). EC were grown to confluence in flat-bottomed 48-well trays. 51Cr-labeled DC (Amersham, UK) were co-incubated with EC monolayers at 37°C for 1h. At the end non-adherent cells were washed away and adherent cells were solubilized with 0.1 ml of 0.1% SDS and radioactivity was counted in a gamma counter. Results represent % of adherent cells ± SD of three replicates/group.

### Transmigration and reverse transmigration assay

This assay was performed as previously described (Bianchi et al. 1993). In brief, EC were grown to confluence on PVP-free polycarbonate filters (5 mm pore) and mounted on modified Boyden chambers over a nitrocellulose filter. 51Cr-labeled DC were seeded in the upper compartment and co-incubated with EC monolayers for 1h at 37°C. Non adherent cells were gently washed away, the radioactivity present in the double filter and in the lower compartment were the transmigrated cells; the adherent cells were considered to comprise both cells bound to EC as well as those that had transmigrated.

In the reverse transmigration assay an upper policarbonate filter was coated with ECM, and the lower policarbonate filter was coated by a monolayer of EC, placed upside down, and mounted in the Boyden chamber. In this assay, the radioactivity present in the lower compartment only accounted for the transmigrated cells. In some experiments policarbonate transwell inserts (5 mm pore, Corning, Costar, Cambridge, MA,USA) were used. Inserts were coated with EC monolayers; chemoattractants, usually 100ng/ml, were seeded in the lower compartment.

### *Acknowledgements*

Supported in part by the National Research Council (Finalized Project Biotec), and by MURST 40% fund (Project Tumori). The Italian Association for Cancer Research (AIRC), is gratefully acknowledged We are indebt to Sanofi, Labège, F, and Novartis, Milan, I for their generous gift of IL-13 and GM-CSF, respectively.

### *References*

Allavena, P., C. Paganin, I. Martin Padura, G. Peri, M. Gaboli, E. Dejana, P. C. Marchisio, and A. Mantovani. (1991). Molecules and structures involved in the adhesion of natural killer cells to vascular endothelium. J. Exp. Med. 173:439–448.

Austyn, J. M. (1996). New insights into the mobilization and phagocytic activity of dendritic cells. J. Exp. Med. 183:1287–1292.

Baggiolini, M., B. Dewald, and B. Moser. (1997). Human chemokines: An update. Annu. Rev. Immunol. 15:675–705.

Banchereau, J. and R. M. Steinman. (1998). Dendritic cells and the control of immunity. Nature 392:245–252.

Barker, C. F. and R. E. Billingham. (1968). The role of afferent lymphatics in the rejection of skin homografts. J. Exp. Med. 128:197–221.

Bianchi, G., M. Sironi, E. Ghibaudi, C. Selvaggini, M. Elices, P. Allavena, and A. Mantovani. (1993). Migration of natural killer cells across endothelial cell monolayers. J. Immunol. 151:5135–5144.

Brand, U., I. Bellinghausen, A. H. Enk, H. Jonuleit, D. Becker, J. Knop, and J. Saloga. (1998). Influence of ECM proteins on the development of cultured human DC. Eur. J. Immunol. 28:1673–1680.

Cella, M., D. Scheidegger, K. Palmerlehmann, P. Lane, A. Lanzavecchia, and G. Alber. (1996). Ligation of CD40 on dendritic cells triggers production of high levels of interleukin-12 and enhances T cell stimulatory capacity: T-T help via APC activation. J. Exp. Med. 184:747–752.

Cyster, J. G. (1999). Chemokines and the homing of dendritic cells to the T cell areas of lymphoid organs. J. Exp. Med. 189:447–450.

D'Amico, G., G. Bianchi, S. Bernasconi, L. Bersani, L. Piemonti, S. Sozzani, A. Mantovani, and P. Allavena. (1998). Adhesion, transendothelial migration, and reverse transmigration of in vitro cultured dendritic cells. Blood. 92:207–214.

De Smedt, T., B. Pajak, E. Muraille, L. Lespagnard, E. Heinen, P. Debaetselier, J. Urbain, O. Leo, and M. Moser. (1996). Regulation of dendritic cell numbers and maturation by lipopolysaccharide in vivo. J. Exp. Med. 184:1413–1424.

Dieu, M. C., B. Vanbervliet, A. Vicari, J-M. Bridon, E. Oldham, S. Ait-Yahia, F. Briere, A. Zlotnik, S. Lebecque, and C. Caux. (1998). Selective recruitment of immature and mature dendritic cells by distinct chemokines expressed in different anatomic sites. J. Exp. Med. 188:373–386.

Frey, J. R. and P. Wenk. (1957). Experimental studies on the pathogenesis of contact eczema in the guinea pig. Arch. Allergy Appl. Immunol. 11:81–100.

Godiska, R., D. Chantry, C. J. Raport, S. Sozzani, P. Allavena, D. Leviten, A. Mantovani, and P. W. Gray. (1997). Human macrophage derived chemokine (MDC) a novel chemoattractant for monocytes, monocyte derived dendritic cells, and natural killer cells. J. Exp. Med. 185:1595–1604.

Greaves, D. R., W. Wang, D. J. Dairaghi, M. C. Dieu, B. de Saint-Vis, K. Franz-Bacon, D. Rossi, C. Caux, T. McClanahan, S. Gordon, A. Zlotnik, and T. J. Schall. (1997). CCR6, a CC chemokine receptor that interacts with macrophage inflammatory protein 3 alpha and is highly expressed in human dendritic cells. J. Exp. Med. 186:837–844.

Gunn, M. D., S. Kyuwa, C. Tam, T. Kakiuchi, A. Matsuzawa, L. T. Williams, and H. Nakano. (1999). Mice lacking expression of secondary lymphoid organ chemokine have defects in lymphocyte homing and dendritic cell localization. J. Exp. Med. 189:451–460.

Hart, D. N. J. (1997). Dendritic cells: unique leukocyte populations which control the primary immune response. Blood 90:3245–3286.

Jancic, C., H. E. Chuluyan, A. Morelli, A. Larregina, E. Kolkoswki, M. Saracco, M. Barboza, W.S. Leiva, and L. Fainboim. (1998). Interactions of dendritic cells with fibronectin and endothelial cells. Immunology 95:283–290.

Lin, C. L., R. M. Suri, R. A. Rahdon, J. M. Austyn, and J. A. Roake. (1998). Dendritic cell chemotaxis and transendothelial migration are induced by distinct chemokines and are regulated on maturation. Eur. J. Immunol. 28:4114–4122.

Macatonia, S. E., S. C. Knight, A. J. Edwards, S. Griffiths, and Fryer P.. (1987). Localization of antigen on lymph node dendritic cells after exposure to the contact sensitizer fluorescein isothiocyanate. Functional and morphological studies. J. Exp. Med. 166:1654–1667.

Macpherson, G. G., C. D. Jenkins, M. J. Stein, and C. Edwards. (1995). Endotoxin-mediated dendritic cell release from the intestine – Characterization of released dendritic cells and TNF dependence. J. Immunol. 154:1317–1322.

Mantovani, A., P. Allavena, A. Vecchi, and S. Sozzani. (1998). Chemokines and chemokine receptors during activation and deactivation of monocytes and dendritic cells and in amplification of Th1 versus Th2 responses. Int. J. Clin. Lab. Res. 28:77–82.

Matsuno, K., T. Ezaki, S. Kudo, and Y. Uehara. (1996). A life stage of particle-laden rat dendritic cells in vivo: their terminal division, active phagocytosis, and translocation from the liver to the draining lymph. J. Exp. Med. 183:1865–1878.

McWilliam, A. S., D. Nelson, J. A. Thomas, and P. G. Holt. (1994). Rapid dendritic cell recruitment is a hallmark of the acute inflammatory response at mucosal surfaces. J. Exp. Med. 179:1331–1336.

McWilliam, A. S., S. Napoli, A. M. Marsh, F. L. Pemper, D. J. Nelson, C. L. Pimm, P. A. Stumbles, T. N. C. Wells, and P. G. Holt. (1996). Dendritic cells are recruited into the airway epithelium during the inflammatory response to a broad spectrum of stimuli. J. Exp. Med. 184:2429–2432.

Ngo, V. N., H. L. Tang, and J. G. Cyster. (1998). Epstein-Barr virus-induced molecule 1 ligand chemokine is expressed by dendritic cells in lymphoid tissues and strongly attracts naive T cells and activated B cells. J. Exp. Med. 188:181–191.

Ngo, V. N., H. Korner, M. D. Gunn, K. N. Scmidt, D. S. Riminton, M. D. Cooper, J. L. Browning, J. D. Sedgwick, and J. G. Cyster. (1999). Lymphotoxin alpha/beta and tumor necrosis factor are required for stromal cell expression of homing chemokines in B and T cell areas of the spleen. J. Exp. Med. 189:403–412.

Piemonti, L., S. Bernasconi, W. Luini, Z. Trobonjaca, A. Minty, P. Allavena, and A. Mantovani. (1995). IL-13 supports differentiation of dendritic cells from circulating precursors in concert with GM-CSF. Eur. Cytokine Netw. 6:245–252.

Power, C. A., D. J. Church, A. Meyer, S. Alouani, A. E. I. Proudfoot, I. Clark-Lewis, S. Sozzani, A. Mantovani, and T. N. C. Wells. (1997). Cloning and characterization of a specific receptor for the novel CC chemokine MIP-3 alpha from lung dendritic cells. J. Exp. Med. 186:825–835.

Randolph, G. J., S. Beaulieu, S. Lebecque, R. M. Steinman, and W. A. Muller. (1998). Differentiation of monocytes into dendritic cells in a model of transendothelial trafficking. Science 282:480–483.

Roake, J. A., A. S. Rao, P. J. Morris, C. P. Larsen, D. F. Hankins, and J. M. Austyn. (1995). Dendritic cell loss from nonlymphoid tissues after systemic administration of lipopolysaccharide, tumor necrosis factor, and interleukin 1. J. Exp. Med. 181:2237–2247.

Rollins, B. J. (1997). Chemokines. Blood 90:909–928.

Saeki, H., A. M. Moore, M. J. Brown, and S. T. Hwang. (1999). Cutting Edge: secondary lymphoid-tissue chemokine (SLC) and CC chemokine receptor 7 (CCR7) participate in the emigration pathway of mature dendritic cells from the skin to regional lymph nodes. J. Immunol. 162:2472–2475.

Sallusto, F., P. Schaerli, P. Loetscher, C. Schaniel, D. Lenig, C. R. Mackay, S. Qin, and A. Lanzavecchia. (1998). Rapid and coordinated switch in chemokine receptor expression during dendritic cell maturation. Eur. J. Immunol. 28:2760–2769.

Sallusto, F. and A. Lanzavecchia. (1999). Mobilizing dendritic cells for tolerance, priming, and chronic inflammation. J. Exp. Med. 189:611–614.

Sironi, M., F. Breviario, P. Proserpio, A. Biondi, A. Vecchi, J. Van Damme, E. Dejana, and A. Mantovani. (1989). IL-1 stimulates IL-6 production in endothelial cells. J. Immunol. 142:549–553.

Sozzani, S., F. Sallusto, W. Luini, D. Zhou, L. Piemonti, P. Allavena, J. Van Damme, S. Valitutti, A. Lanzavecchia, and A. Mantovani, (1995). Migration of dendritic cells in response to formyl peptides, C5a and a distinct set of chemokines. J. Immunol. 155:3292–3295.

Sozzani, S., W. Luini, A. Borsatti, N. Polentarutti, D. Zhou, L. Piemonti, G. D'Amico, C. A. Power, T. N. Wells, M. Gobbi, P. Allavena, and A. Mantovani. (1997). Receptor expression and responsiveness of human dendritic cells to a defined set of CC and CXC chemokines. J. Immunol. 159:1993–2000.

Sozzani, S., P. Allavena, G. D'Amico, W. Luini, G. Bianchi, M. Kataura, T. Imai, O. Yoshie, R. Bonecchi, and A. Mantovani. (1998). Cutting edge: Differential regulation of chemokine receptors during dendritic cell maturation: A model for their trafficking properties. J. Immunol. 161:1083–1086.

Sozzani, S., P. Allavena, A. Vecchi, and A. Mantovani. (1999). The role of chemokines in the regulation of dendritic cell trafficking.J. Leukoc. Biol. 1999. 66:1-9.

Steinman, R. M. (1991). The dendritic cell system and its role in immunogenicity. Annu. Rev. Immunol. 9:271–296.

Wang, B., H. Fujisawa, L. Zhuang, S. Kondo, G. M. Shivji, C. S. Kim, T. W. Mak, and D. N. Sauder. (1997). Depressed Langerhans cell migration and reduced contact hypersensitivity response in mice lacking TNF receptor p75. J. Exp. Med. 159:6148–6155.

Willimann, K., D. F. Legler, M. Loetscher, R. Stuber Roos, M. B. Delgado, I. Clark-Lewis, M. Baggiolini, and B. Moser. (1998). The chemokine SLC is expressed in T cell areas of lymph nodes and mucosal lymphoid tissues and attracts activated T cells via CCR7. Eur. J. Immunol. 28:2025–2034.

Yoshida, R., T. Imai, K. Hieshima, J. Kusuda, M. Baba, M. Kitaura, M. Nishimura, M. Kakizaki, H. Nomiyama, and O. Yoshie. (1997). Molecular cloning of a novel human CC chemokine EBI1-ligand chemokine that is a specific functional ligand for EBI1, CCR7. J. Biol. Chem. 272:13803–13809.

